# Comminuted Paraspinal Rib Fractures with Resultant Impending Penetrating Aortic Injury Requiring Costovertebral Rib Fixation: A Case Report

**DOI:** 10.3390/medicina60122063

**Published:** 2024-12-15

**Authors:** Soon-Ki Min, Tae-Seok Jeong, Yang-Bin Jeon

**Affiliations:** 1 Department of Trauma Surgery, Gachon University Gil Medical Center, Incheon 21565, Republic of Korea; minsk0611@gilhospital.com; 2 Department of Traumatology, Gachon University College of Medicine, Incheon 21565, Republic of Korea; jeongtaeseok@gilhospital.com

**Keywords:** rib fracture, costovertebral rib fixation, costotransverse plate fixation, blunt thoracic trauma

## Abstract

*Background and Objectives*: Rib fractures are common in patients with trauma, and patients with multiple rib fractures often require surgical stabilization. Because rib fractures may occur at different sites along the ribs, the technical approach to surgical stabilization varies. Here, we present a case of posterior rib fractures with multiple paraspinal fragmented rib segments that were successfully treated with costovertebral plate fixation. *Case Presentation*: A truck driver was injured after falling from the top of a truck. Computed tomography scans of the chest showed multiple flail segments along the paraspinal and posterolateral regions with a clinically evident flail chest. Owing to the proximity of the flail segments to the thoracic spine, rib plating was performed across the ribs and the transverse processes of the thoracic spine with the assistance of a neurosurgeon. The patient was extubated on postoperative day 1 and discharged successfully after the other traumatic injuries were treated. *Discussion*: Far posterior rib fractures close to the spine may be challenging, particularly if plates for rib fractures cannot be placed on the ribs alone. For such challenges, costotransverse plating is a feasible surgical option. However, the anatomical orientation of the rib and the transverse process of the thoracic spine are different, which complicates surgical planning and maneuvers. Therefore, a thorough understanding of the costotransverse anatomy is critical for successful surgical stabilization of fractured ribs. *Conclusions*: This is a good example of a challenging case of rib fractures requiring paraspinal plate stabilization.

## 1. Introduction

Rib fractures are common, accounting for approximately 20% of blunt thoracic trauma cases and 10% of all trauma cases [1,2]. Patients with rib fractures can present with mild to severe symptoms that often require surgical intervention. Although the mainstays of rib fracture management are pain control, pulmonary toilet, and ventilatory support when needed, surgical stabilization of rib fractures is often required [1,3]. The treatment modalities for rib fractures are categorized largely into two types: internal support and external support. The internal support includes positive-pressure ventilation to internally stabilize the rib cage, and it is still widely utilized to stabilize the costal structure and support the respiratory function. The external support includes both non-operative and operative measures. Non-operative measures such as straps, external splints, and traction devices like forceps percutaneously fixed to the chest wall are rarely used today. Operative measures include open reduction and internal fixation, which has evolved from simply throwing sutures and wrapping wires around the fractured ribs to the current plating system using metal plates fixed along the fractured ribs with screws [4,5]. The surgical stabilization of rib fractures is continuously gaining support because it reduces the ventilator duration and length of intensive care unit (ICU) admission, thus resulting in fewer respiratory complications and less economic burden [3,6]. Indications for surgical stabilization of rib fractures include flail chest, severe respiratory compromise requiring ventilatory support, injured or threatened internal organs, and severe pain [1,7].

Rib fractures, whenever present, are indicative of high-energy trauma. Therefore, they are often accompanied by concomitant injuries, including other thoracic injuries such as pneumothorax, hemothorax, and pulmonary contusions [8]. In addition, traumatic aortic injury is one of the most concerning injuries among thoracic traumas. However, those afflicted by rib fractures are very rare [9,10,11,12]. Left posterior rib fractures, when displaced, may have sharp edges facing towards the descending aorta, which possibly leads to rib fracture-related aortic injuries, and these injuries may manifest at different times after traumatic events with varying fatality rates. In particular, left proximal rib fractures near the spine not only pose potential threats to the aorta but also present challenges for rib plating due to insufficient screw landing zones for stabilization [9]. 

Here, we present a case of left comminuted paraspinal rib fractures with flail characteristics and displacements threatening the descending aorta, which requires difficult surgical planning.

## 2. Case Presentation

**Figure 1:**
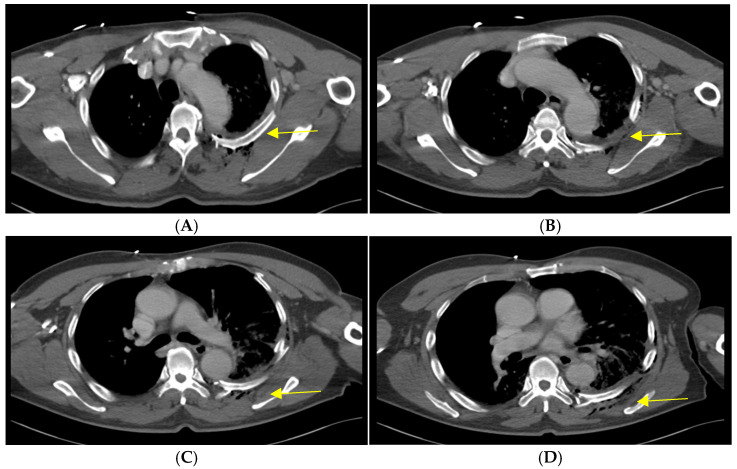
Axial views of the initial chest CT scans showing the most displaced left-sided fractured ribs (arrow). The CT images are shown in sequential order from the 4th rib to the 7th rib (**A**–**D**). (**C**) The tip of the fractured 6th rib poses an imminent threat to the aorta.

A 64-year-old truck driver fell from the top of a dump truck, estimated to be approximately 3 m high. When the emergency medical service arrived at the scene of the accident, the patient was lying flat on the ground, complaining of chest pain and dyspnea. The initial vital signs were as follows: blood pressure of 100/60 mmHg, pulse rate of 98 beats/min, respiratory rate of 25 breaths/min, and oxygen saturation of 88%. Oxygen was supplied at a rate of 15 L/min with a partial rebreather facial mask, which improved the oxygen saturation to 96%. The patient was transferred to the nearest trauma center. The vital signs measured on arrival at the trauma center were as follows: blood pressure of 125/97 mmHg; pulse rate of 95 beats/min; respiratory rate of 32 breaths/min; and oxygen saturation of 92%. Initial laboratory results showed a hemoglobin level of 12.3 g/dL, a white blood cell count of 11350 per μL, and a lactate level of 2.4 mmol/L, indicative of mild impairment of oxygen delivery. Whole-body computed tomography (WBCT) scan was performed, revealing initial diagnoses, including minor traumatic brain injury with skull fractures and scalp laceration, left clavicle fracture, multiple left-sided rib fractures with ipsilateral hemopneumothorax and pulmonary contusion, and left kidney injury. Rib fractures were observed at the 2nd to the 12th ribs, with moderate to severe displacements. Notably, the displaced 5th and 6th left ribs were positioned such that they nearly damaged the descending thoracic aorta (Figure 1). 

**Figure 2:**
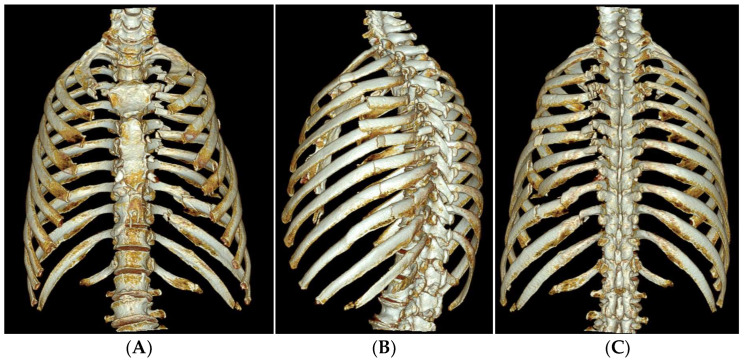
3D reconstructed images of the rib CT taken on HD #2 for operative planning. Anteroposterior (**A**), left oblique (**B**), and posteroanterior (**C**) views of the 3D reconstruction images show a radiological flail chest with posterior paraspinal fractured fragments.

Due to the significant pulmonary contusion and dyspnea, the patient’s oxygen saturation dropped below 90% after WBCT was taken, and he was intubated and initially placed on ventilatory support. A thoracic trauma surgeon was consulted due to the radiologic flail chest, resultant respiratory compromise, and displaced posterior rib segments threatening the patient’s descending aorta. Early surgical stabilization was scheduled. The following day, rib CT was performed for surgical planning (Figure 2), and the patient was taken to the operating room for surgical stabilization of the rib fractures. 

**Figure 3:**
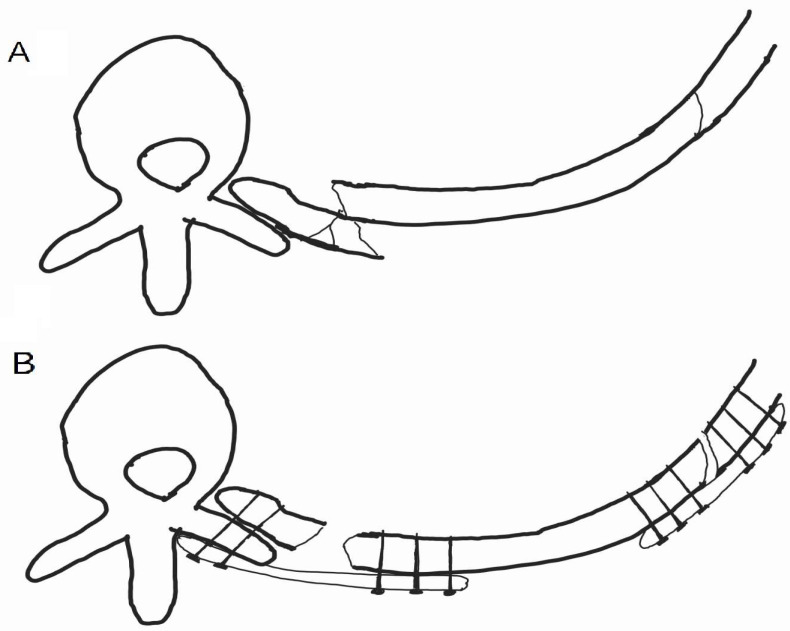
A graphical representation of SSRF for this case. (**A**) The medial rib close to the spine was comminuted and displaced with sharp edges facing towards the descending aorta. (**B**) The comminuted fragments were removed, and the broken rib medial segments were approximated as much as possible. However, due to muscular attachments along the rib, a floating portion could not be amended, and medial approximation, in turn, resulted in a dehiscence of the lateral fracture site.

**Figure 4:**
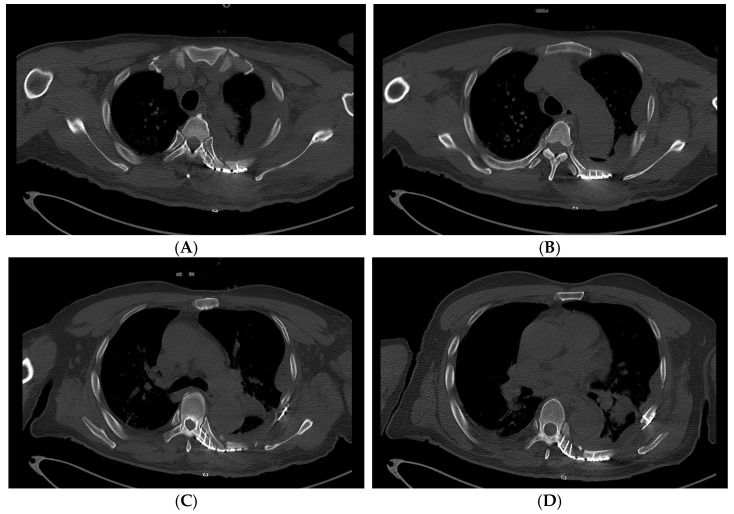
Axial view images of the post-operative rib CT showing costovertebral plating. The previously displaced 6th rib fragment (**C**) threatening the aorta is now aligned along with other flail segments (4th–7th, **A**–**D**).

**Figure 5:**
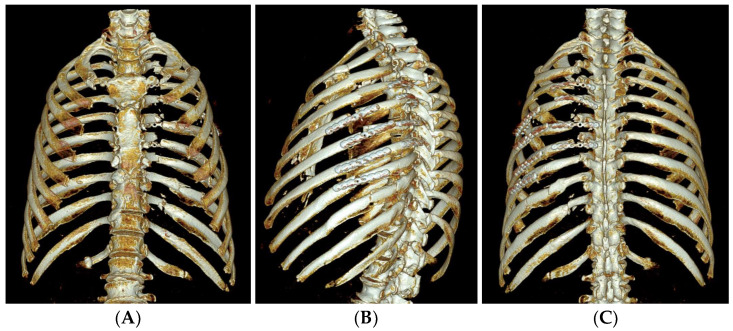
The 3D reconstructed images of the rib CT taken after rib fixation. Anteroposterior (**A**), left oblique (**B**), and posteroanterior (**C**) views of the 3D reconstruction images show successfully plated rib fractures.

**Figure 6:**
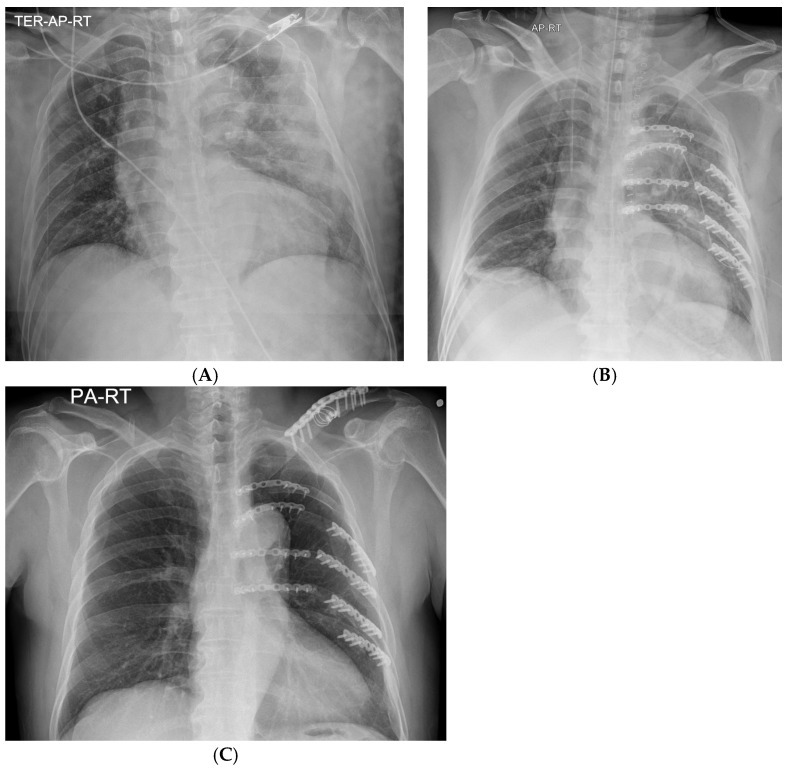
Plain chest radiographs taken (**A**) on the admission day, (**B**) immediately after surgical stabilization of rib fractures, and (**C**) on outpatient follow-up.

**Figure 7:**
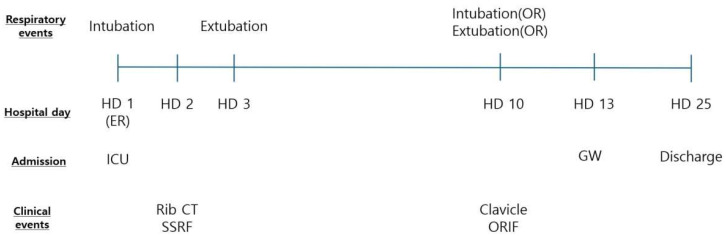
The timeline of the clinical course of the patient.

A longitudinal incision was made along the left border of the thoracic spine. Because the posteromedial fracture lines were very close to the spine and the short, fractured segments were adjacent to each other, it was impossible to secure the plate landing zones on the ribs. Therefore, the short segments were removed to decrease the risk of organ injury. With the help of a spine surgeon, rib plating (JEIL ARIX rib system, Jeil Medical, Seoul, Republic of Korea) was performed on the 4th to 7th ribs, with the medial sides of the plates anchored on the transverse processes of the thoracic spine. However, the removed segments left a space even after an effort at approximation, and the plates were placed across short floating spaces, which measured approximately 1.2 cm (Figure 3). Subsequently, a separate oblique incision was made, and the posterolateral aspects of the fractured 5th to 8th rib segments were plated for further stability and to attenuate tensile stress on the medial plates along floating portions (Figure 3, Figure 4 and Figure 5). The lung and descending aorta were inspected through a thoracotomy, and no injury was evident other than lung contusions. The pleural cavity was thoroughly irrigated, and a chest tube was placed for the management of hemopneumothorax. The following day, the patient was weaned from the mechanical ventilator and extubated. The high-flow nasal cannula was initially applied for respiratory support and was slowly tapered according to the patient’s respiratory status and ability, and the chest tube was removed on postoperative day 8. The patient required one additional general anesthesia for clavicular fracture fixation on the 10th hospital day (HD) but did not require mechanical ventilatory support post-operation. The patient was discharged from the ICU on HD 13 without clinical and pathological respiratory complications such as pneumonia and was discharged from the hospital on HD 25 after post-operative management. The patient reported minor chest pain, and a chest X-ray taken during an outpatient follow-up after discharge showed no complications (Figure 6). Figure 7 presents a timeline of the clinical hospital course of the patient.

## 3. Discussion

This case is a good example of successful early surgical stabilization of rib fractures, which otherwise might have led to early or delayed aortic injury, as evidenced by previous reports [11,12]. In addition, early surgical intervention led to expedited escape from mechanical ventilation and significantly decreased injury-associated and ventilator-associated respiratory complications.

A literature search revealed scant information on paraspinal rib fractures presenting with insufficient screw-landing zones on the ribs. Schuette et al., Bartscherer et al., and Zhao et al. reported cases in which costotransverse rib plating was performed [2,9,10]. However, this case is different in that the proximal parts of the posterior ribs showed multiple fracture points in extreme proximity to the spine, and very short, displaced segments were present. Visual inspection of the displaced segments of the 4th–7th ribs showed that they were too short and fragile, and screw fixation was not an option. Therefore, these segments were removed, and the open ends of the remaining segments were smoothed with a rongeur and pulled closer for plating. Although the ribs were plated as closely as possible, floating portions were observed in the areas where fragments were found. Plates were positioned across the floating portions, and medial approximation of the ribs resulted in dehiscence at the lateral fracture points (Figure 3). As a result, the posterolateral areas of the fractured ribs were plated to ensure stability and to attenuate tensile stress along the ribs, which may have been applied solely on the medial plates.

The anatomical relationship between the rib and transverse process varies and is complex at different spinal levels. The first costotransverse joint is oriented nearly parallel to the thoracic spine, and the subsequent costotransverse joints are gradually tilted such that the ventral side of the costal tubercle faces more cranially. Hence, a thorough understanding of the anatomical relationship between the rib and transverse processes is critical for exposing the surgical area and for proper surgical planning for costotransverse plating [13]. Moreover, since there is no commercial plate available for the unique costovertebral anatomy, careful shaping of currently available plates is essential for successful alignment and stabilization.

Most current rib plating systems incorporate a bicortical system in which screws need to be fixed across both the anterior and posterior cortices of the bone to sustain plate stability for maximal results. However, bicortical systems can be concerning in cases when screws are placed too deeply in a way that they pose threats to internal structures. Such a problem is more concerning when plating posteromedial ribs near the descending aorta. Currently, there is only one rib plating system available in South Korea, and it is a domestically manufactured bicortical system. Although having the screws penetrate both cortices of the bone provides plate stability, measuring and selecting the proper size of screws may be challenging for an anatomically complex area such as in this case. Furthermore, sharp edges of the screws may be exposed internally, which may cause internal organ injuries and discomfort. In such instances, a commercially available unicortical rib system may be more beneficial.

This case, which required a detailed understanding of the thoracic and spinal anatomies, exemplifies the importance of good cooperation between a thoracic trauma surgeon and a neurotrauma surgeon. It presents a surgical option for managing difficult cases. However, due to the complex anatomic relationship between the ribs and spinal structures and the continuously mobile nature of the rib cage due to respiration, long-term outcomes need to be studied, and the potential applicability of unicortical systems warrants further investigation. 

## 4. Conclusions

Posterior paraspinal rib fractures, especially left-sided rib fractures, may threaten vital organs in the thoracic cavity. When indicated for surgical stabilization, these rib fractures may require a costotransverse approach. Considering their anatomical complexity, thorough planning and consultation with a neurosurgeon are critical for desirable outcomes.

## Data Availability

The datasets used or analyzed during the current study are available from the corresponding author upon reasonable request.
